# Linking Pain Sensation to the Autonomic Nervous System: The Role of the Anterior Cingulate and Periaqueductal Gray Resting-State Networks

**DOI:** 10.3389/fnins.2020.00147

**Published:** 2020-02-27

**Authors:** David Johannes Hohenschurz-Schmidt, Giovanni Calcagnini, Ottavia Dipasquale, Jade B. Jackson, Sonia Medina, Owen O’Daly, Jonathan O’Muircheartaigh, Alfonso de Lara Rubio, Steven C. R. Williams, Stephen B. McMahon, Elena Makovac, Matthew A. Howard

**Affiliations:** ^1^Department of Neuroimaging, King’s College London, London, United Kingdom; ^2^Department of Surgery and Cancer, Imperial College London, London, United Kingdom; ^3^Department of Technology and Health, Italian National Institute of Health, Rome, Italy; ^4^Wolfson Centre for Age Related Diseases, King’s College London, London, United Kingdom; ^5^Sackler Institute for Translational Neurodevelopment, King’s College London, London, United Kingdom; ^6^Centre for the Developing Brain, King’s College London, London, United Kingdom; ^7^MRC Centre for Neurodevelopmental Disorders, King’s College London, London, United Kingdom

**Keywords:** pain, autonomic nervous system, heart rate variability, fMRI, resting state, periaqueductal gray, anterior cingulate cortex

## Abstract

There are bi-directional interactions between the autonomic nervous system (ANS) and pain. This is likely underpinned by a substantial overlap between brain areas of the central autonomic network and areas involved in pain processing and modulation. To date, however, relatively little is known about the neuronal substrates of the ANS-pain association. Here, we acquired resting state fMRI scans in 21 healthy subjects at rest and during tonic noxious cold stimulation. As indicators of autonomic function, we examined how heart rate variability (HRV) frequency measures were influenced by tonic noxious stimulation and how these variables related to participants’ pain perception and to brain functional connectivity in regions known to play a role in both ANS regulation and pain perception, namely the right dorsal anterior cingulate cortex (dACC) and periaqueductal gray (PAG). Our findings support a role of the cardiac ANS in brain connectivity during pain, linking functional connections of the dACC and PAG with measurements of low frequency (LF)-HRV. In particular, we identified a three-way relationship between the ANS, cortical brain networks known to underpin pain processing, and participants’ subjectively reported pain experiences. LF-HRV both at rest and during pain correlated with functional connectivity between the seed regions and other cortical areas including the right dorsolateral prefrontal cortex (dlPFC), left anterior insula (AI), and the precuneus. Our findings link cardiovascular autonomic parameters to brain activity changes involved in the elaboration of nociceptive information, thus beginning to elucidate underlying brain mechanisms associated with the reciprocal relationship between autonomic and pain-related systems.

## Introduction

Neural networks involved in pain processing are intimately linked to the autonomic nervous system (ANS) (Benarroch, 2006): On the one hand, the body’s response to pain is defined by changes in ANS parameters (Kyle and McNeil, 2014); on the other, alterations in autonomic arousal can also influence the experience of pain (Terkelsen et al., 2004). There is growing interest in mindfulness-based and other mind-body interventions in the treatment and management of pain (Stanos, 2012; Goyal et al., 2014), with changes in autonomic balance one of the likely underlying mechanisms of action (Tang et al., 2015). Whilst various regions of the central nervous system (CNS) are known to play a role in both pain and the ANS, currently there is a lack of knowledge concerning how pain-autonomic interactions may be reflected by functional connections in the brain.

One possible mechanism underlying this pain-autonomic interaction is the baroreflex, the negative feedback loop used to maintain stable blood-pressure (Suarez-Roca et al., 2018). This mechanism has been associated with observed reduction in pain perception in healthy controls during spontaneous high blood pressure (during which baroreceptors are activated) and also during mechanical stimulation of baroreceptors (Edwards et al., 2001; Duschek et al., 2007; Reyes del Paso et al., 2014). Decreased baroreceptor sensitivity has also been described in some chronic pain conditions (Davydov et al., 2018).

Heart rate variability (HRV), which is derived from variations in interval length between consecutive heart beats (Task Force, 1996; Berntson et al., 1997; Thayer et al., 2010a, 2012), is often estimated to assess the autonomic response to experimental pain (Koenig et al., 2014). Low frequency (LF)-HRV spectral power is thought to represent the baroreflex-dependent outflow to the heart, whereas high frequency (HF) is interpreted as indicator of vagal cardiac control, and the ratio between the two measures (LF/HF) as a reflection of sympathovagal balance (Pagani et al., 1986, 2012; Montano et al., 2009; Thayer et al., 2010a; Goldstein et al., 2011; Reyes del Paso et al., 2013). Experimentally induced pain increases LF power and the LF/HF ratio (Koenig et al., 2014), indicating an increased engagement of the baroreflex. Few studies have employed tonic cold pain stimuli, but results from cold water hand immersion tests (i.e., the cold pressor test) point toward a similar tendency of increased LF-HRV (Mourot et al., 2009; Streff et al., 2010).

A pain-suppressive effect associated with larger HF spectral power at baseline has been found (Nahman-Averbuch et al., 2016; Tracy et al., 2018), which might be explained by superior capacity to engage vagal cardiac control (Reyes del Paso et al., 2011; Busch et al., 2013; Zunhammer et al., 2013; Tracy et al., 2018). Conversely, higher resting LF-HRV has been found to predict reduced thermal pain sensitivity (Appelhans and Luecken, 2008; Tracy et al., 2018). Patients with chronic pain conditions such as fibromyalgia often show reduced HF power in addition to increased LF and LF/HF, suggesting dysregulated autonomic cardiac control (Meeus et al., 2013) and altered baroreflex engagement (Bruehl et al., 2017). In brief, HRV indices respond to noxious stimuli and relate to the subjective intensity of pain, but can also predict the experience of pain, indicating a bi-directional association where nociception influences the ANS and, conversely, the ANS modulates the experience of pain.

Modern neuroimaging techniques, such as functional magnetic resonance imaging (fMRI) and positron emission tomography (PET), have been used to investigate the brain regions associated with autonomic activity during various tasks and conditions. Significant correlations between HRV, amygdala and medial prefrontal cortex (mPFC) activity have been demonstrated (Thayer et al., 2012; Beissner et al., 2013; Steinfurth et al., 2018). In resting-state fMRI (rs-fMRI) paradigms, HRV measures were associated with functional connectivity of different resting state networks (RSNs). For example, Jennings et al. (2016) found participants’ HF-HRV at rest to correlate with resting state connectivity of the mPFC, but not with salience (SN) or default mode networks (DMN). Functional connectivity of the dorsal anterior cingulate cortex (dACC) to the thalamus and brainstem co-varies with HF-HRV, whilst LF-HRV relates to dACC connectivity with the temporoparietal junction (Chang et al., 2013). Further, measures of vagal output have been shown to be associated with functional connectivity between cortical areas and parts of the brainstem (e.g., Smith et al., 2015; Bär et al., 2016).

Many brain regions involved in ANS activity are also active during the experience of pain, including the anterior cingulate cortex (ACC), amygdala, and periaqueductal gray (PAG) (Leone et al., 2006; Heinricher and Fields, 2013). The dACC is consistently found to be involved in the CNS response to noxious stimulation (Vogt et al., 2003; Apkarian et al., 2005; Leone et al., 2006; Duerden and Albanese, 2013; Jensen et al., 2016). Whilst the more rostral parts are associated with the affective component of pain, the most dorsal aspect (bordering the mid-cingulate cortex) encodes the objective aspects of pain (e.g., stimulus intensity) (Rainville et al., 1999; Singer et al., 2004; Wager et al., 2004; Amodio and Frith, 2006; Boccard et al., 2014). Taken together, the dACC forms part of a network of brain regions involved in the detection of salient sensory events, including the multimodal context-dependent experience of pain, but is also intimately linked to the ANS and the sensing of internal body states (i.e., interoception) (Craig, 2002; Tracey and Mantyh, 2007; Legrain et al., 2011). Similarly, the midbrain PAG receives both peripheral nociceptive input and descending projections from the hypothalamus, amygdala, and rostral ACC. Providing output to medullary centers, the PAG is an essential component of a descending pain modulatory system that inhibits or facilitates nociceptive processing within the spinal dorsal horn (Ossipov et al., 2010; Heinricher and Fields, 2013). PAG neurons projecting to autonomic centers in the medulla are also involved in cardiovascular changes observed during opioid-dependent and independent endogenous analgesia (Benarroch, 2006; Green et al., 2006). In addition, stimulation of the PAG alters baroreflex sensitivity and cardiac control (Pelosi et al., 2007; Pereira et al., 2010; Benarroch, 2012; Lagatta et al., 2016).

Due to extensive functional overlap between structures involved in autonomic control, nociception, and pain sensation at different levels of the spinal cord, brainstem, midbrain, and cortex (Benarroch, 2001, 2006), the reciprocal ability of the ANS to modulate nociceptive information is not surprising. Amongst others, emotional appraisal (Wiech and Tracey, 2009), attention (James and Hardardottir, 2002; Bradshaw et al., 2012), mood (Carter et al., 2002), hypnotic suggestion (Rainville et al., 1999), and stress (Terkelsen et al., 2004; Ballegaard et al., 2014) can influence the subjective pain experience. Any of these states corresponds with changes in ANS activity (Wiech et al., 2008; Kreibig, 2010) and baroreflex sensitivity (Fejes et al., 2020).

Whilst previous studies have described brain networks underlying pain-induced sympathetic reactions in response to tonic pain (Kobuch et al., 2017, 2018), only one study so far has investigated how variations in HRV relate to brainstem functional connectivity changes observed during a prolonged painful experience: Sclocco et al. (2016) convolved HRV with the hemodynamic response function during 6 min of pressure pain, identifying several brainstem nuclei (specifically, rostral ventromedial medulla, ventral nucleus reticularis/nucleus ambiguous, and pontine nuclei) associated with pain-evoked HRV alterations. To date, the bi-directional association between pain-engaged higher brain networks (involving, for example, the anterior cingulate cortex) and HRV (either resting or in response to pain) remains unexplored.

In this study, we combined sampling of HRV and rs-fMRI, both during rest and tonic noxious cold stimulation, in a group of healthy participants. We adopted a hypothesis-driven region of interest (ROI) approach, drawing upon regions known to play important roles in both ANS function and nociceptive processing, namely the dACC and the PAG (Benarroch, 2006; Leone et al., 2006; Tracey and Mantyh, 2007; Legrain et al., 2011). Following previous studies, we hypothesized that HRV measures at baseline and during noxious stimulation will be associated with subjective pain intensity ratings. Further, we hypothesized that PAG and dACC RSNs would be affected by a tonic noxious stimulus, and that these pain-induced rs-fMRI changes would correlate with HRV parameters and subjective ratings of pain. Lastly, we explored whether baseline HRV is associated with the brain response to a noxious stimulus.

## Materials and Methods

### Participants

Twenty three healthy participants took part in the experiment. Two participants were discarded due to poor quality of the physiology data. The final sample comprised 21 participants (8 females, mean age: 26.1, SD: 5.2). Further, pain ratings were obtained from 17 of those participants only (five participants did not attend the final testing session). All participants were right-handed as assessed by the Edinburgh handedness inventory (Oldfield, 1971). In addition to MRI contraindications, exclusion criteria (verified by means of standardized questionnaires or semi-structured interview) included: a history of psychiatric illness; substance abuse [as verified by Sections 11 and 12 of the Schedules for Clinical Assessment in Neuropsychiatry (SCAN) (Wing et al., 1990); and by the Alcohol Use Disorders Identification Test (AUDIT) (Higgins-Biddle and Babor, 2018)]; chronic pain conditions; diagnosed medical or psychological conditions that might compromise participation in the study or interfere with somatosensation; cardiovascular medication and medication which might affect temperature sensitivity (e.g., tricyclic antidepressants). All female participants were tested within the follicular phase to reduce hormonal effects on HRV (Sato et al., 1995) and pain sensitivity (Martin, 2009; Iacovides et al., 2015).

To minimize the influence of diurnal variations on pain responses (Strian et al., 1989; Hodkinson et al., 2014), rs-fMRI networks activity (Blautzik et al., 2013; Jiang et al., 2016), and HRV (Ewing et al., 1991; Li et al., 2011; Xhyheri et al., 2012), participants were always tested at the same time of the day. At the beginning of each visit, participants were tested for drugs of abuse (urine drug test) and alcohol consumption (alcohol breathalyzer). Participants’ autonomic reactions (blood pressure and heart rate) were tested at the beginning and at the end of each experimental session, in a sitting and standing position, to identify any anomalous cardiovascular behavior. All participants provided written informed consent. The study was approved by the King’s College Research Ethics Committee in accordance with the principles of the Helsinki declaration.

### Experimental Procedure

Participants attended three visits: a first familiarization session, a scanning session, and a post-scanning session. During the familiarization session, participants were accustomed with the cold stimulation and the MRI environment.

During the scanning session, participants underwent three consecutive resting-state fMRI blocks, each of 6 min duration: a baseline resting condition (“Baseline”), and a prolonged noxious cold stimulation (“Cold-pain”) and a post-cold recovery session. For this study, data from the first two blocks were analyzed, whilst the results of the last block are presented in Makovac et al. (2019). To elicit pain, 2°C cold-water was circulated via a custom-made aluminum thermode (4 × 20 cm) applied to the volar surface of the participants’ left forearm, via a high capacity (700W) solid state circulating chiller unit (Thermotek RC22A750) employed to deliver stable temperature control of the afferent stimulation over time. Following an initial stabilization period (∼20 s), a mean temperature was maintained at 2.5°C (±0.9°C) throughout the experimental block.

During each resting state period, participants were instructed to rest with their eyes open, and focus on a fixation cross presented at the center of the screen, without thinking of anything and not falling asleep. Heart rate (HR) was sampled continuously during the Baseline and Cold-pain condition by means of photoplethysmography (PPG), using an inbuilt MRI-compatible pulse oximeter (General Electric) fitted to the participants’ right index finger. In stationary conditions, pulse oximetry has been shown to be a good surrogate measure for ECG-derived HRV (Gil et al., 2010; Schäfer and Vagedes, 2013).

In our original experimental paradigm, the “cold-pain” resting state session was followed immediately by a further “post-cold” session (see Makovac et al., 2019), which precluded the provision of an intermediate subjective response to the prolonged noxious stimulation. To circumvent this, each participant’s subjective experience of cold pain was explored in a further post-scanning session. Here, participants were instructed to rate the pain level and unpleasantness experienced during the same 6-min 2°C cold stimulation on a visual analog scale (VAS) ranging from 0 (no pain) to 100 (worst pain imaginable) (Howard et al., 2006; Marquand et al., 2010). Cold pain ratings were robust and reliable (see Supplementary Material S1 for more details).

### Heart Rate Variability

Photoplethysmography inter-beat interval (IBI) data were plotted in Matlab, visually inspected, and potential artifacts removed manually. IBI values were used as inputs into Kubios HRV Standard ver. 3.0.2 software (Tarvainen et al., 2014). Detrending was performed based on smoothness priors. Frequency domain measures were extracted into MS Excel and IBM SPSS Statistics for Macintosh (ver. 24) for statistical analysis. Data were then scanned for outliers using boxplots and exploratory statistics in SPSS. Values for HRV frequency bands are LF: 0.04–0.15 Hz, and HF: 0.15–0.4 Hz, as recommended by a 1996 task force publication on HRV (Task Force, 1996). The natural logarithms of LF and HF power (in ms^2^) were calculated, with the aim to reduce skewness and kurtosis of HRV parameters and to enable the data to more closely conform to the assumptions of normality. Other studies have reported that the log of LF power correlates positively with the log of baroreflex-cardiovagal gain (Moak et al., 2007; Rahman et al., 2011).

In order to differentiate early HRV alterations from later ANS habituation to noxious stimulation, HRV frequency measures were extracted from two separate intervals; Interval 1 (0–3 min from the beginning of the session) and Interval 2 (3–6 min), separately for the Baseline and Cold-pain session. HRV measures derived from a 3-min sample have good inter-session reliability (see Supplementary Material S3).

### MRI Acquisition and Preprocessing

MR images were acquired on a 3T GE MR750 scanner, with a 32-channel receive-only head coil (NovaMedical). Structural volumes were obtained using the high-resolution three-dimensional magnetization-prepared rapid gradient-echo sequence (TR = 7312 ms, TE = 3.02 ms, flip angle = 11°, slice thickness = 1.2 mm, 196 sagittal slices, FOV = 270 mm). Functional datasets used T2^∗^weighted multi-echo imaging (EPI) sensitive to blood oxygenation level dependent (BOLD) signal (TR = 2 s, TE1 = 12 ms, TE2 = 28 ms; TE3 = 44 ms; flip-angle 80°, 32 slices, 3 mm slice thickness, 240 mm FOV, voxel size 3.75 × 3.75 × 3 mm). By acquiring multiple echo images per slice, multi-echo fMRI allows to identify non-BOLD related sources of signal and preserves the signal of interest (Dipasquale et al., 2017).

Pre-processing was performed using AFNI (Cox, 1996), the Advanced Normalization Tools (ANTs) (Avants et al., 2011), and FSL (Smith et al., 2004). Steps included volume re-alignment, time-series de-spiking and slice time correction. After pre-processing, functional data were optimally combined (OC) by taking a weighted summation of the three echoes using an exponential T2^∗^ weighting approach (Posse et al., 1999). The OC data were then de-noised adopting a Multi-Echo ICA approach implemented by the tool meica. py (Version v2.5 beta9) (Kundu et al., 2013, 2014), given its effectiveness in removing physiological and motion-related noise and increasing temporal SNR (Kundu et al., 2013; Dipasquale et al., 2017). Briefly, multi-echo principal component analysis was first used to reduce the data dimensionality in the OC dataset. Spatial ICA was then applied to one echo, and the independent component time-series were fitted to the pre-processed time-series from each of the three echoes to generate ICA weights for each echo. These weights were then fitted to the linear TE-dependence and TE-independence models to generate F-statistics and component-level κ and ρ values, which, respectively, indicate BOLD and non-BOLD weightings. The ρ metrics were then used to identify non-BOLD-like components to be regressed out of the OC dataset as noise. For further technical details on multi-echo ICA refer to Kundu et al. (2015).

Next, white matter and cerebrospinal fluid time-series were regressed out using FSL. A high-pass temporal filter with a cut-off frequency of 0.005 Hz was applied, and the data were spatially smoothed with a 5 mm FWHM Gaussian kernel. Each participant’s dataset was co-registered to its corresponding structural scan using affine-only registration. Then, using a non-linear registration approach, functional data were normalized to standard MNI152 space and resampled to 2 × 2 × 2 mm^3^ using ANTs.

### Statistical Analyses

#### HRV Reactions to Pain

We tested for a difference in HRV parameters either with pain induction or between the initial and final interval of each test condition. A two-way within-subject ANOVA was used to explore the main effect of Condition (Baseline, Cold-pain), Interval (starting 3 min, final 3 min), and the Condition x Interval interaction, separately for LF-HRV and HF-HRV.

Next, we examined whether subjects’ pain sensitivity correlated with HRV at baseline and during cold stimulation. All data were expressed as means (±SD). Differences at *p* < 0.05 were regarded as significant. Data analysis was performed with SPSS 23.0 for Windows (SPSS Inc., United States).

### Seed-Based fMRI Analysis

Anatomical ROIs were constructed using the Marsbar toolbox implemented in SPM 12^1^. The cingulate-seed was located at MNI_xyz_ = (2, 8, 38) (7 mm spherical radius; right dACC) and the PAG ROI at MNI_xyz_ = (0, −30, −1) (3 mm spherical radius). Our ROIs were based on results from pre-existing data acquired by our group (Makovac et al., 2019) and previously published examples of the dACC (Kong et al., 2010a; Duerden and Albanese, 2013; Wilcox et al., 2015) and the PAG (Kong et al., 2010b; Zyloney et al., 2010; Mainero et al., 2011). Functionally, we chose these two regions because of their known involvement in both pain processing and ANS control (see Supplementary Material S4 for a Neurosynth – https://neurosynth.org/ – based meta-analysis of pain-related and ANS-related studies, S4). It is of note that, given the extension of the sphere used to build our ROI, the dACC ROI covered an area on the boundary between dACC and medial cingulate cortex (MCC), as defined by Vogt (2016).

The average resting state fMRI time-series in each ROI was extracted for each participant and for each scan and used as a regressor at 1st level SPM analysis with the purpose of determining the voxels in the brain showing a significant correlation with each ROI. Next, group analyses were performed, in which participants’ first level contrast images for the Baseline and Cold-pain conditions were included in a paired *t*-test, to explore the network of areas positively associated with our seed regions in each condition.

We performed three different regression analyses, using HRV measures and subjective pain ratings as covariates of interest. First, we explored the relationship between our resting-state networks and pain-induced HRV alterations. We tested whether baseline dACC and PAG functional connectivity predicted HRV changes during pain, and whether dACC and PAG functional connectivity during cold pain was associated with HRV reactions to cold pain. Next, we investigated whether baseline dACC-PAG functional connectivity underlies the association between HRV and pain. Here, both HRV and subjective pain ratings were entered into the same general linear model. Lastly, to explore the modulatory role of the HRV toward pain perception, we tested whether baseline HRV measures could predict pain-related dACC-PAG RSN alterations.

Due to potential gender-specific differences in pain processing (Moulton et al., 2006; Henderson et al., 2008; Paller et al., 2009), gender was used as a covariate of no interest. Similarly, age has been shown to influence HRV in healthy individuals (O’Brien et al., 1986; Antelmi et al., 2004) and was thus controlled for in our analyses. Statistical threshold was set to *p* < 0.05 – FWE-corrected at cluster level (cluster size defined using uncorrected voxel-level threshold *p* < 0.005), according to Gaussian Random Field Theory (Worsley et al., 1992).

## Results

### Sample Characteristics

Table 1 provides a summary of the main demographic and baseline characteristics of our sample. After the 6-min cold stimulation, participants gave an average pain rating of 45.8 (SD = 22.2) on a 0–100 VAS scale.

**TABLE 1:** Sample characteristics and heart rate variability (HRV) measures.

**Total *n***	**21**					
**Sample characteristic**	**M**	**SD**	**HRV measure**		**M**	**SD**
	
**age**	26.1	(±5.2)	**Log LF** (absolute) in ms^2^			
**gender ratio**				*BL*	2.913 (1461.12)	±0.405 (±1195.53)
*female. n(%)*	8	38.1		*Cold-pain*	2.936 (1537.21)	±0.460 (±1686.35)
**BMI**	22.1	(±2.4)	**Log HF** (absolute) in ms^2^			
**rSBP** *mnHg*	118.8	(±9.6)		*BL*	2.938 (1468.86)	±0.403 (±1577.27)
**HRBL** *bpm*	52.7	(±10)		*Cold-pain*	3.022 (1713.5)	±0.382 (±1654.64)
**HRCold-pain** *bpm*	50.8	(±9.9)				
**VAS** (*n* = 17)	45.8	(±22.2)	**LF/HF**			
**caffeine** *drinks p.d.*	1.7	(±1.3)		*BL*	−0.0247	(±0.426)
**Cigarettes** *p.d.*	0.5	(±2.3)		*Cold-pain*	−0.0863	(±0.333)
**alc.** *units p. week*	3.2	(±4.8)				

*Alc, Alcohol consumption; BL, baseline; BMI, body mass index; average HR, heart rate; HF, LF, High- and Low-frequency; and their ratio, LF/HF in log-normalized and absolute values, average VAS, visual analog scale rating of 2°C cold-pain stimulation, rSBP, resting systolic blood pressure; M, Mean values as well as SD, standard deviations are reported. p.d., per day; p.w., per week; Bpm, beats per minute.*

### The Effect of Cold Pain on HRV Measures

We found an overall increase in logLF-HRV from Baseline to Cold-pain [mean (SD) = 2.91 (0.41) and 2.94 (0.46), respectively; *F*_(__1_,_17__)_ = 7.79, *p* = 0.013]. Whether the HRV data was sampled during the first or second half (interval) of each experimental condition did not affect this result [*F*_(__1_,_17__)_ = 1.39, *p* = 0.25] and there was no significant difference between intervals across conditions (*F* < 1). As regards logHF-HRV, we did not observe a significant difference between Baseline and Cold-pain conditions (*F* < 1), nor was there an effect of Interval (*F* < 1) or a Condition × Interval interaction effect (*F* < 1).

### Correlation Between HRV Measures and Pain Ratings

HRV as measured during any of the experimental conditions was not associated with subjective cold pain intensity ratings (*p* > 0.05 for both logLF-HRV and logHF-HRV).

### Seed-Based Resting-State fMRI Results

#### Identification of Baseline Resting State Networks

##### dACC-seed

**FIGURE 1:**
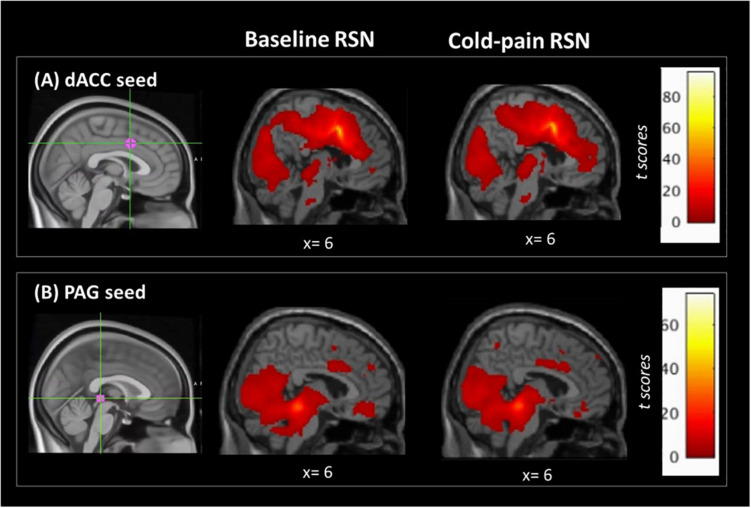
Regions of interest used in the seed-based analysis and their associated RSNs at baseline and during Cold-pain. **(A)** dACC ROI, MNI_xyz_ = (2, 8, 38). This ROI had a 7 mm spherical radius. Anatomically, the dACC-seed lies in Brodmann area 24. **(B)** PAG ROI, MNI_xyz_ = (0, –30, –1), the size was 3 mm spherical radius, and the seed was positioned in the anatomical midline. ROIs (in magenta) are overlaid on MNI-standardized T1-weighted images for visualization purposes. Color bars indicate *t* scores.

The dACC ROI was functionally connected with clusters in the left insula, bilateral superior frontal, precentral and postcentral gyri, the precuneus and the posterior cingulate cortex (Figure 1A and Table 2).

**TABLE 2:** Resting state networks associated with the PAG and dACC seed regions.

		**Cluster**		**Voxel**	
**Contrast**	**Brain area**	***k***	***p FWE***	***T (F)***	***MNI xyz***
*Positive association with PAG seed*					
	Left hippocampus	217110	<0.001	17.30	−14 −18 −18
	Right hippocampus			14.08	22 −14 −22
	Posterior cingulate cortex			13.46	20 −42 −2
	Thalamus			9.37	12 −36 −2
	Cerebellum			9.33	2 −52 −12
*Positive association with dACC seed*					
	Middle cingulate cortex	921610	<0.001	32.52	0 −6 36
	Left Insula			25.04	−30 20 0
	Superior frontal gyrus			19.65	24 −4 52
	Posterior cingulate cortex/Precuneus			18.75	18 −40 42
	Parietal operculum			18.56	52 −32 20
*FC changes during cold pain- dACC seed*					
	Superior forntal gyrus	588	0.001	4.66	−12 50 18
	Anterior cingulate cortex			4.32	−8 38 4
	Frontal lobe			4.00	−12 46 30

*PAG, periaqueductal gray; ACC, anterior cingulate cortex; FC, functional connectivity.*

##### PAG-seed

The resting network of our PAG-seed consisted of a cluster in bilateral hippocampus, posterior cingulate cortex, thalamus and the cerebellum as well as ventro-medial prefrontal cortex (Figure 1B and Table 2).

#### The Effect of Cold-Pain on Resting-State Networks

**FIGURE 2:**
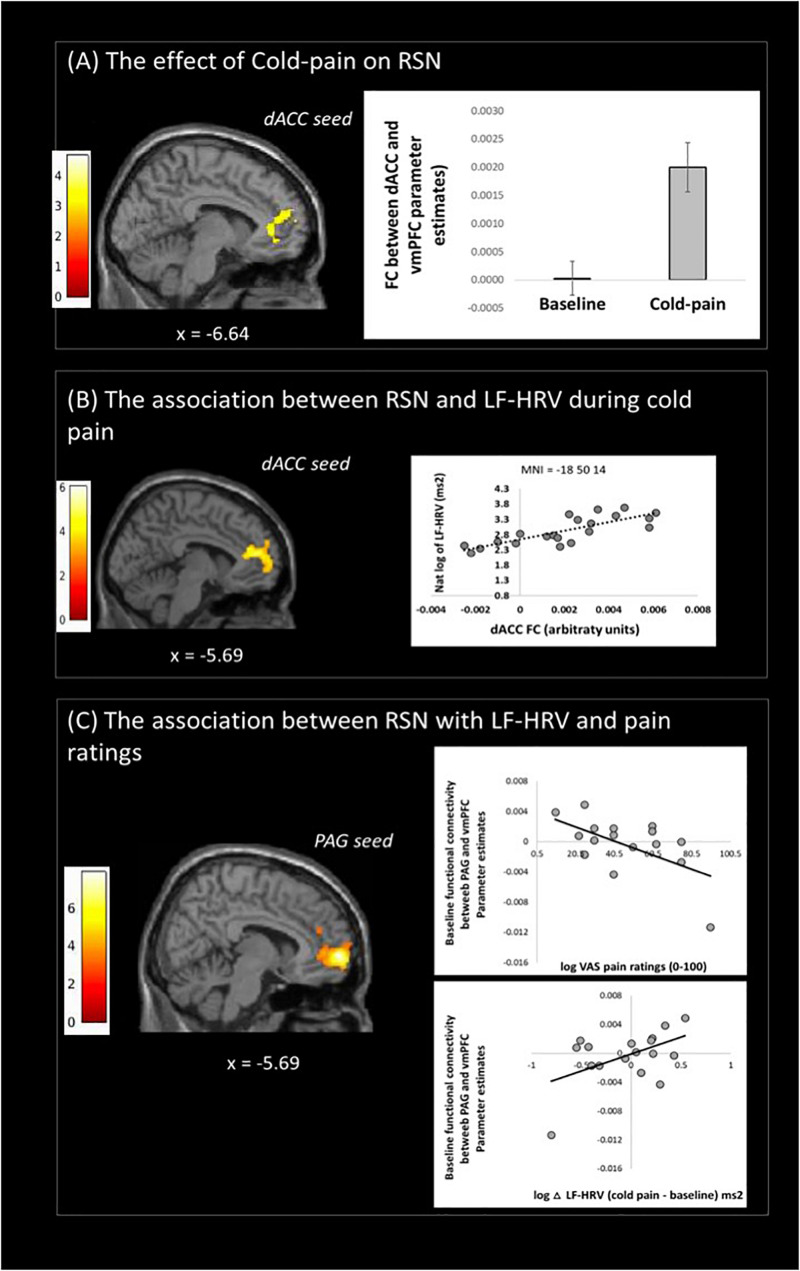
Resting state fMRI results: **(A)** changes of dACC resting state network (RSN) with cold-pain stimulation, **(B)** Changes in dACC RSN associated with logLF-HRV as measured during cold-pain, **(C)** Baseline PAG functional connectivity with the vmPFC was associated with both logLF-HRV during cold-pain and participants’ pain ratings (VAS). Color bars indicate t scores.

Functional connectivity of the dACC increased in the Cold-pain condition with clusters in the contralateral rostral ACC, superior frontal gyrus, and the frontal pole (Figure 2A and Table 2). Upon noxious cold stimulation, functional connectivity of the PAG with the precuneus increased [results reported in Makovac et al. (2019)].

#### The Association Between RSNs and Pain-Related HRV Alterations

During Cold-pain, a positive association was observed between logLF-HRV and the functional connectivity between dACC and vmPFC (Figure 2B). We did not observe any significant association between PAG functional connectivity and pain-induced HRV alterations.

#### The Inter-Relationship Between RSNs, Pain-Related HRV Alterations and Pain Ratings

We tested whether baseline functional connectivity with PAG predicts both pain-related HRV reactions and pain ratings. Baseline functional connectivity between PAG and vmPFC expressed a significant logLF-HRV × cold pain ratings interaction. This effect was driven principally by the co-expression of a positive correlation with logLF-HRV during cold pain and a negative correlation with pain ratings. Thus, stronger baseline PAG-vmPFC connectivity was associated on one hand with stronger autonomic reaction during cold pain and on the other hand with lower subjective perception of cold pain (Figure 2C).

### Baseline HRV as Predictor of Functional Connectivity Changes to Cold Pain

**FIGURE 3:**
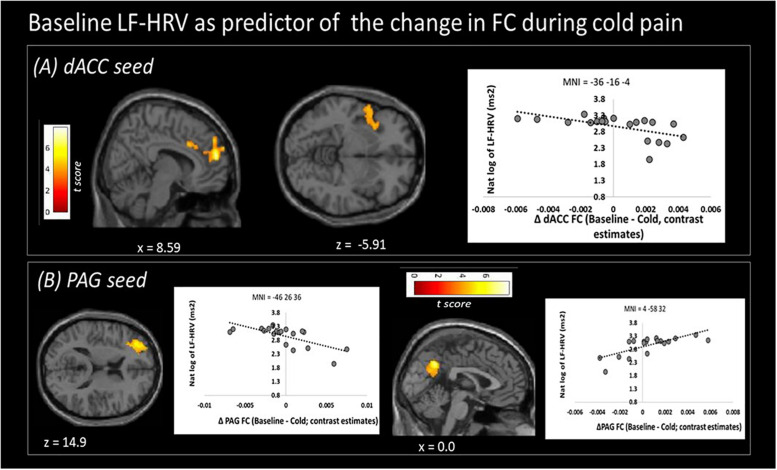
Low frequency heart rate variability (LF-HRV) at baseline predicts functional connectivity changes (Δ FC) of both seed regions upon cold-pain stimulation. **(A)** Baseline logLF-HRV predicted a decrease in dACC functional connectivity with regions in the right dorsolateral prefrontal area, frontal pole, and the right central opercular cortex. **(B)** Baseline logLF-HRV predicted a decrease during cold pain in functional connectivity between the PAG and right dorsolateral prefrontal cortex and an increase between the PAG and the precuneus. Color bars indicate t scores.

Higher baseline logLF-HRV measures predicted decreases in functional connectivity between dACC and superior frontal gyrus/dorsolateral prefrontal areas and between dACC and left AI (Figure 3A and Table 3) during cold pain. Baseline logLF-HRV values predicted an increase in functional connectivity between the PAG^2^ and precuneus, and a decrease between the PAG and the right dorso-lateral prefrontal cortex (Figure 3B and Table 3).

**TABLE 3:** Brain areas showing an association with HRV and cold pain.

		**Cluster**				**Voxel**
	**Brain area**	***k***	***p FWE***	***Side***	***T (F)***	***MNI*_xyz_**
**(1)**	**Association between dACC functional connectivity and LF-HRV during cold-pain**					
	Medial prefrontal cortex	2296	0.000	L	6.04	−18 50 14
						6 64 12
						2 48 20
**(2)**	**Association between baseline PAG functional connectivity and both LF-HRV during cold-pain and cold pain ratings**					
	Medial prefrontal cortex	2659	0.000	B	7.87	8 62 −2
**(3)**	**Baseline LF-HRV as a predictor of functional connectivity changes during cold-pain**					
**3.a)**	**dACC seed**					
	Superior frontal gyrus	825	0.001	R	6.8	10 56 22
	Frontal pole					24 56 22
	Dorsolateral prefrontal cortex					6 66 28
	Anterior insula	539		L		−36 16 −4
						−50 12 −10
						−40 2 −2
**b)**	**PAG seed**					
	Precuneus	941	0.194	B	6.55	4 −58 2
						−10 −56 6
						−22 −60 28
	Dorsolateral prefrontal cortex	1353	0.251	L	6.33	−46 26 36
						−44 34 28
						−20 58 −10

## Discussion

The aim of this study was to examine the relationship between HRV and brain functional connectivity during painful experimental stimulation. Specifically, we investigated the effect of a tonic noxious cold stimulus in a group of healthy participants, implementing simultaneous rs-fMRI and HRV sampling. Our results support a role of ANS activity, as indexed by HRV, in brain connectivity during pain and specify functional connections of our dACC and PAG seed regions that are associated with measurements of LF-HRV. In particular, we identified a three-way relationship between HRV, cortical brain networks known to underpin pain processing, and participants’ subjectively reported pain experiences. Baseline PAG-vmPFC functional connectivity was associated with higher LF-HRV during cold stimulation and lower subjective cold pain ratings, suggesting that the role of the ANS in the modulation of nociception might relate (at least in part) to PAG-cortical functional connections.

Our findings combine two separate streams of research: the association between LF-HRV and pain and the role of PAG functional connectivity in pain perception. Higher baroreflex activation and parasympathetic activity (indexed by LF and HF-HRV) prior to and during noxious stimulation is associated with reduced pain intensity or higher pain thresholds (Duschek et al., 2007; Appelhans and Luecken, 2008; Nahman-Averbuch et al., 2016; Tracy et al., 2018). In addition to the well-established role of the PAG in descending nociceptive modulation (Ossipov et al., 2010; Benarroch, 2012), PAG-mPFC connectivity (Sprenger et al., 2011) and activity of the mPFC alone (Bogdanov et al., 2015) have been linked to the efficacy of endogenous analgesic mechanisms and to vagal cardiac control (Critchley et al., 2011). A recent brainstem-focused fMRI study showed that subjective pain intensity is not only influenced by the reaction of the brain to a noxious stimulus, but also by the connectivity of the PAG prior to stimulation (Stroman et al., 2018). These results indicate that the PAG is functionally associated with the hypothalamus and several brainstem areas involved in autonomic regulation, highlighting the potential importance of homeostatic autonomic control in the descending modulation of nociception. Our findings provide direct support for this theory. Meta-analytical evidence shows that right mPFC is associated with HRV during both emotional and cognitive/motor tasks (Thayer et al., 2012). Others have reported an association between mPFC resting state connectivity and HRV, often linked to emotion regulation. Our findings expand this work by showing that the functional communication between the PAG and the mPFC is associated with LF-HRV reactions to noxious stimulation as well as the subjective perception of cold pain intensity.

During noxious cold stimulation, functional connectivity of the right dACC with the mPFC was positively correlated with LF-HRV. A reduction in the dACC-(ventral)mPFC functional connectivity has been shown to partially mediate heart rate increases during socially stressing experiences (Wager et al., 2009). Whilst we did not formally assess mediation effects, our finding extends this notion to include pain, an experience which requires both emotional and physiological regulation. In more general terms, this finding underlines the known role of the mPFC in context appraisal and autonomic adaptation, not least in situations of threat (Schiller et al., 2008; Thayer et al., 2012).

Higher baseline LF-HRV, which is often considered as a measure of baroreflex activation (Goldstein et al., 2011), was associated with an increase in PAG-precuneus functional connectivity during tonic noxious stimulation. Areas of the DMN (including the precuneus) show reduced functional connectivity during pain (Baliki et al., 2008; Kong et al., 2010a). In view of the opinion of higher LF-HRV as a likely anti-nociceptive mechanism, sustained connectivity between the PAG and the precuneus is thus consistent with a reduced nociception-associated brain response. A potential underlying mechanism is offered by Kucyi et al. (2013), who associated greater functional connectivity between PAG and precuneus/mPFC with participants’ tendencies to disengage their attention from the noxious stimulation (“mind wandering”), thus achieving pain relief by means of distraction (Sprenger et al., 2012). Possibly, effective engagement of descending pain modulation as associated with LF-HRV allows for, or is part of, participants’ natural tendencies to “take their minds off the pain.” Accordingly, this finding offers preliminary support for theories of a protective role of mind wandering in optimizing states of bodily arousal during challenges to homeostasis (Baars, 2010; Ottaviani et al., 2013).

Higher baseline LF-HRV was also associated with a stronger decrease in functional connectivity between both the PAG and dACC seeds with the dlPFC. DlPFC activity is commonly reported in pain experiments. Functionally part of the central executive-control network (CEN), the dlPFC shows increased activation during the performance of cognitively demanding tasks (Seeley et al., 2007) and is associated with cognitive difficulties seen in chronic pain (Bushnell et al., 2013). Albeit based on experimentally induced pain, our results suggest a link between cognitive and homeostatic systems, and indicate a possible common neuronal substrate for cognitive difficulties (Apkarian et al., 2004; Bushnell et al., 2013) and HRV alterations (Meeus et al., 2013) often reported in chronic patients. Functional connectivity of the dACC with the dlPFC has previously been shown to co-vary with LF-HRV (Chang et al., 2013), supporting a role of the ANS in this functional link.

Higher baseline resting LF-HRV also related to decreases in functional connectivity between the dACC and the left AI during noxious stimulation. Activity of the insular cortex is frequently coupled to that of the cingulate cortex. Together, they form part of an interoceptive network which facilitates emotion and self-awareness but also modulates autonomic function (Craig, 2002). A specific role in cardiovascular control has been postulated (Nagai et al., 2010). Importantly, insular activity is associated with top down suppression of baroreflex activity induced by a stress challenge (Gianaros et al., 2012). We suggest that the ANS-associated connectivity between AI and dACC plays a central role in interoception during pain and might also contribute to baroreflex-induced anti-nociception (as reviewed by Suarez-Roca et al., 2018). Also, activity in the AI is commonly seen during pain (Legrain et al., 2011). More specifically, the AI is involved in the cognitive evaluation of pain (Brooks et al., 2002; Wiech et al., 2010; Segerdahl et al., 2015). Furthermore, combined AI and ACC activation was found to relate to the emotional components of pain, such as psychological pain, and empathy for a loved one in pain (Singer et al., 2004; Immordino-Yang et al., 2009). Less functional connectivity between the dACC and AI with high baseline LF-HRV is thus consistent with maintained baroreflex activity during noxious stimulation, and reduced activity in an interoceptive and cognitive/emotional key area of pain perception.

Our data provide reason for further investigation into the role of autonomic cardiovascular modulation in central network dynamics. The mPFC and the precuneus are considered part of the DMN (Fox and Raichle, 2007), whilst the dlPFC is a core node in the CEN (Seeley et al., 2007). The dACC and the AI, on the other hand, form the salience network (SN) which responds to the subjective salience of cognitive, emotional, and homeostatic stimuli. The SN is thought to play a critical role in switching between the off-task DMN and the on-task CEN (Sridharan et al., 2008; Menon and Uddin, 2010). Both DMN and CEN alterations have been described in chronic pain states (Baliki et al., 2008; Cauda et al., 2009; Malinen et al., 2010; Napadow et al., 2010, 2012; Tagliazucchi et al., 2010; Bolwerk et al., 2013; Jiang et al., 2016; Alshelh et al., 2018; Androulakis et al., 2018). Here we demonstrated that by phenotyping participants based on LF-HRV, a physiological variable, we were able to predict changes in components of these functional networks during the experience of a noxious stimulus. Albeit not having assessed network dynamics explicitly, these findings suggest that the ANS may be implicated in the salience network-mediated switch from default to central cognitive-executive modes during the experience of tonic experimental pain. Speculatively, a pronounced LF-HRV at baseline (interpreted as a more pronounced engagement of the baroreflex) might predict a reduced tendency for a SN-mediated switch of network dynamics toward an executive mode, whilst LF-HRV during the experience of pain might be associated with maintained DMN dynamics. It remains to be determined whether these initial neurophysiological findings apply in patients with chronic pain.

Finally, hierarchical interactions between cognitive, emotional and autonomic processes are also a quintessential component of the neurovisceral integration (NVI) model, as proposed by Thayer and Lane (2000). This model postulates that cardiac vagal tone (i.e., the contribution of the parasympathetic nervous system to cardiac regulation) is an indicator of the functional balance between neural networks involved in the regulation of emotions and cognition (Thayer and Lane, 2000). This Central Autonomic Network (CAN) (Benarroch, 1993) consists of parts of the prefrontal cortex (anterior cingulate, insula, orbitofrontal, and ventromedial cortex), limbic cortex (amygdala and hypothalamus), and brain stem areas (i.e., PAG and ventromedial medulla). Our data fits with this model, as they suggest that the resting cardiac autonomic tone (possibly related to the baroreflex) and the cardiac autonomic reactivity to pain is associated with the functional organization of some pain-related networks (i.e., those related to the dACC and PAG, involving other structures of the CAN such as vmPFC and anterior insula), which in turn is related to the amount of experienced pain. It is of note, however, that our data do not strictly explore the vagal influence on cardiac regulation, but rather the reactivity of the baroreflex, which is more representative of sympatho/vagal balance. Importantly, the NVI model suggests that HRV is a measure of the flexibility of the entire brain-body system, with the view that flexible systems are adaptive and responsive to the environment, allowing for functional oscillations between different states (Smith et al., 2017). In the context of pain, we argue that an individual with an adequate ANS reaction to pain is more efficient in triggering those mechanisms which have the aim of re-establishing adaptive homeostasis (i.e., descending pain modulatory mechanisms). Future studies should aim to explore this model in clinical populations.

We acknowledge some methodological limitations: First, the method of HRV acquisition, photoplethysmography (PPG), is not considered gold standard. Despite this, individual studies such as Gil et al. (2010) show that while subjects are at rest, PPG is a good surrogate measure for ECG-derived HRV. Sufficient accuracy under stationary conditions has also been confirmed by a review on the topic (Schäfer and Vagedes, 2013). Furthermore, the inter-session reliability of our measurements was good (see Supplementary Material S3), providing confidence in our method of capturing HRV. We also acknowledge a relatively small sample size; this study was formally powered to detect the effect of a noxious stimulation on functional brain networks rather than to determine interactions between functional networks and the ANS. Future studies should aim to replicate our findings with larger samples in order to validate the robustness and reliability of our results. Lastly, and this note of caution applies to many studies linking brain activity to function, the associations between HRV, functional connectivity and subjective pain does by no means imply causality. Further studies will have to elucidate if there is a causal relationship between the above findings. Dynamic causal modeling techniques applied to brain connectivity data (Friston, 2009) offer a potential means to conduct these investigations.

In conclusion, we have demonstrated that the engagement of brain regions involved in the cognitive, emotional and limbic processing of pain is intimately linked to autonomic profiles and subjective pain sensitivity. As the first study to explore the association between pain-related HRV and brain functional connectivity, we provide an improved understanding of the relationship between pain perception and autonomic cardiovascular control, likely involving the baroreflex.

Future research should determine whether this functional connectivity is altered in chronic pain, and if modulation of ANS activity might protect from chronic pain. Such data may catalyze the development and utility of ANS-targeted pain treatments, such as HRV biofeedback, vagal stimulation or baroreceptor activation therapies.

## Data Availability Statement

At the date of publication, the datasets for this manuscript are not publicly available because of limited ethical approval to share participant data. Requests to access the datasets should be directed to EM (elena.makovac@kcl.ac.uk).

## Ethics Statement

The studies involving human participants were reviewed and approved by the Ethics Committee King’s College London. The patients/participants provided their written informed consent to participate in this study.

## Author Contributions

DH-S analyzed the data and wrote the manuscript. GC, OD, JJ, SM, OO’D, JO’M, AdLR, SW, and SM contributed substantially to the conception of the experiment and to the analysis of the data and reviewed the manuscript. EM conceived and conducted the experiment and contributed substantially to the writing and revision of the manuscript. MH supervised the team, contributed to the planning and conduction of the experiment, and made major contributions to the revision of the manuscript.

## Conflict of Interest

The authors declare that the research was conducted in the absence of any commercial or financial relationships that could be construed as a potential conflict of interest.

## Footnotes

1 http://marsbar.sourceforge.net/

2 We identified one participant with PAG functional connectivity values outside inter-quartile difference criteria for outlier identification (Tukey, 1977). Accordingly, the participant was excluded from baseline logLF-HRV functional connectivity analyses and the final analysis was performed with 20 participants.
