# Supplementary material for: Linking Pain Sensation to the Autonomic Nervous System: The Role of the Anterior Cingulate and Periaqueductal Gray Resting-State Networks
Source: Front Neurosci. 2020 Feb 27;14:147. doi: 10.3389/fnins.2020.00147 (PMC7527240; doi:10.3389/fnins.2020.00147)
Supplement: Supplementary file 1 [file Data_Sheet_1.docx]

**Supplementary material**

**S1. Reliability of cold-pain stimulation.**

To test the reliability of cold-induced pain; we ran an event-related experiment with alternating periods of cold (2°, 32 sec) and warm stimulations (28°, 40 sec), in two different identical sessions (session A and B). At the end of each cold and warm block, participants were instructed to rate the perceived pain intensity, on a VAS scale ranging from 0 (no pain) to 100 (maximal pain imaginable in the current experimental situation).

ICC was computed using SPSS v19.0 (SPSS Inc., Chicago, IL, USA). ICC values were denoted < 0.4 as poor, 0.4–0.59 as fair, 0.60–0.74 as good, and > 0.75 as excellent (Fleiss, Levin & Paik, 2013). A value of 1.0 indicates near-perfect agreement between the values of the test and retest sessions, a value of 0.0 would indicate that there was no agreement between the values of the test and retest sessions.

Results: Overall, we observed “excellent” degree of within-subject reliability for cold ratings between session 1 and 2 (ICC = 0.78, 95% CI [0.53, 0.91]).

**S2.** **Pilot data exploring the continuous subjective perception of cold pain over a 6-minute stimulating interval.**

**Figure S2.** Participants were instructed to continuously rate their subjective perceived pain intensity (dashed line) during a six-minute cold pain stimulation. Overall, the perception of pain remained stable, with a slight tendency to decrease toward the end of the stimulation, likely representing habituation to the painful stimulation. Average pain ratings (bar graph; mean +/- s.e.m) did not differ significantly when grouped across 1-minute intervals.

**S3. Reliability of HRV measures calculated across 3-minutes intervals**

Heart rate data were acquired during resting state baseline and during 6-minutes cold stimulation, over two identical sessions (with an inter-session interval ranging from 2 weeks to 2 month). Heart rate variability measures were extracted from two intervals during each session (0-3 minutes and 3-6 minutes).

ICC analyses demonstrated good inter-session reliability (ICC = 0.66, 95% CI [0.25, 0.86]) for LF-HRV at rest for the first interval (0-3 minutes) and fair reliability (ICC = 0.58, 95% CI [0.14, 0.83]) for LF-HRV at rest during the second interval (3-6 minutes). Similarly, during cold stimulation, LF-HRV demonstrated good inter-session reliability for the 0-3 minutes interval (ICC = 0.69, 95% CI [0.34, 0.87]), and fair reliability for the LF-HRV values extracted from the 3-6 minutes interval (ICC = 0.45, 95% CI [-0.002, 0.75]).

**
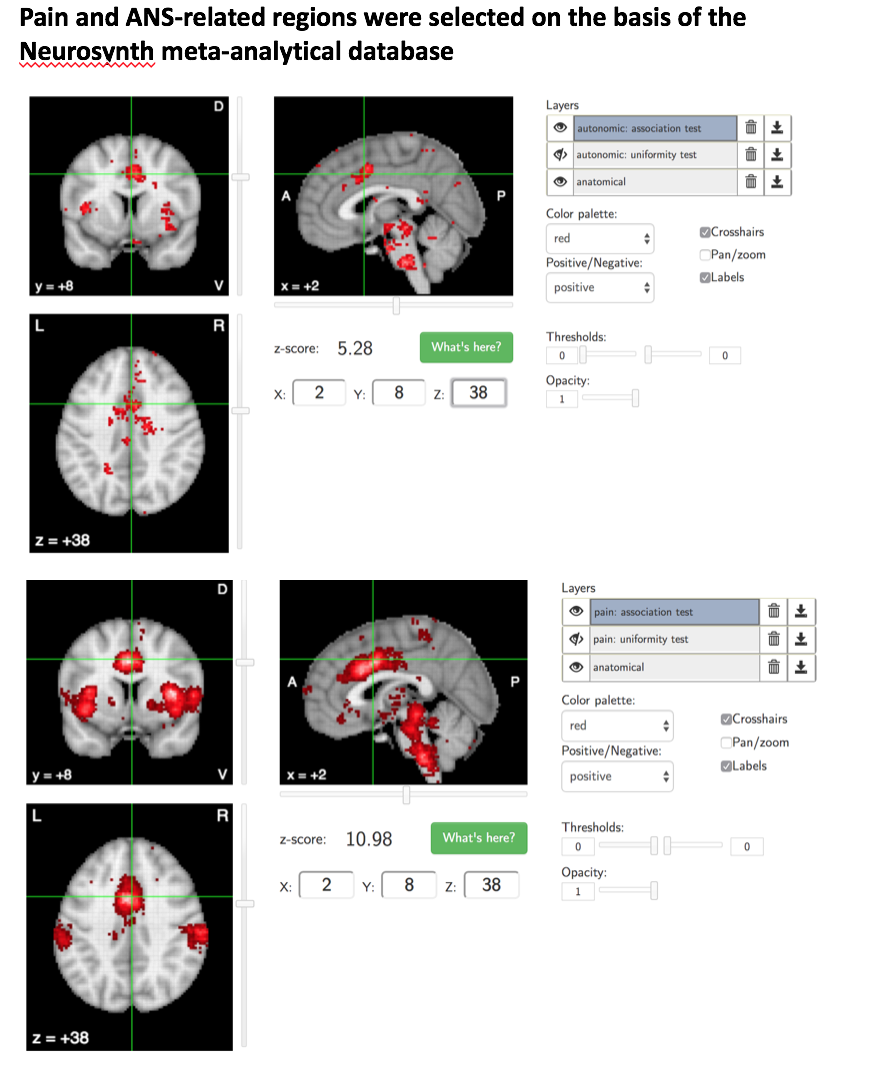
**

**S4.** Neurosynth (<https://neurosynth.org/analyses/)> based automated meta-analysis of studies involved in pain (search word= ‘pain’; n=516) and studies involved in autonomic control and elaboration (search word= ‘autonomic’, n= 117). Both meta-analyses highlight an involvement of the periaqueductal grey (PAG) and of the dorsal anterior cingulate area (dACC), MNI [2 8 38].

**References**

Fleiss, J. L., Levin, B., & Paik, M. C. (2013). *Statistical methods for rates and proportions*: John Wiley & Sons.
